# Evolution of Cocirculating Varicella-Zoster Virus Genotypes during a Chickenpox Outbreak in Guinea-Bissau

**DOI:** 10.1128/JVI.02337-14

**Published:** 2014-12

**Authors:** Daniel P. Depledge, Eleanor R. Gray, Samit Kundu, Samantha Cooray, Anja Poulsen, Peter Aaby, Judith Breuer

**Affiliations:** a Division of Infection and Immunity, University College London, London, United Kingdom; b Bandim Health Project, INDEPTH Network, Bissau, Guinea-Bissau; c Child and Adolescent Clinic, Rigshospitalet, Copenhagen, Denmark; d Research Center for Vitamins and Vaccines (CVIVA), Bandim Health Project, Statens Serum Institut, Copenhagen, Denmark

## Abstract

Varicella-zoster virus (VZV), a double-stranded DNA alphaherpesvirus, is associated with seasonal outbreaks of varicella in nonimmunized populations. Little is known about whether these outbreaks are associated with a single or multiple viral genotypes and whether new mutations rapidly accumulate during transmission. Here, we take advantage of a well-characterized population cohort in Guinea-Bissau and produce a unique set of 23 full-length genome sequences, collected over 7 months from eight households. Comparative sequence analysis reveals that four distinct genotypes cocirculated among the population, three of which were present during the first week of the outbreak, although no patients were coinfected, which indicates that exposure to infectious virus from multiple sources is common during VZV outbreaks. Transmission of VZV was associated with length polymorphisms in the R1 repeat region and the origin of DNA replication. In two cases, these were associated with the formation of distinct lineages and point to the possible coevolution of these loci, despite the lack of any known functional link in VZV or related herpesviruses. We show that these and all other sequenced clade 5 viruses possess a distinct R1 repeat motif that increases the acidity of an ORF11p protein domain and postulate that this has either arisen or been lost following divergence of the major clades. Thus, sequencing of whole VZV genomes collected during an outbreak has provided novel insights into VZV biology, transmission patterns, and (recent) natural history.

**IMPORTANCE** VZV is a highly infectious virus and the causative agent of chickenpox and shingles, the latter being particularly associated with the risk of painful complications. Seasonal outbreaks of chickenpox are very common among young children, yet little is known about the dynamics of the virus during person-to-person to transmission or whether multiple distinct viruses seed and/or cocirculate during an outbreak. In this study, we have sequenced chickenpox viruses from an outbreak in Guinea-Bissau that are supported by detailed epidemiological data. Our data show that multiple different virus strains seeded and were maintained throughout the 6-month outbreak period and that viruses transmitted between individuals accumulated new mutations in specific genomic regions. Of particular interest is the potential coevolution of two distinct parts of the genomes and our calculations of the rate of viral mutation, both of which increase our understanding of how VZV evolves over short periods of time in human populations.

## INTRODUCTION

Varicella-zoster virus (VZV; subfamily alphaherpesvirus), causes chickenpox (varicella), an infection mainly of childhood, and shingles (zoster), a painful dermatomal rash that follows reactivation of latent endogenous virus in sensory ganglia. The virus is transmitted in aerosols, resulting mainly from the rupture of fluid filled skin blisters which are characteristic of both chickenpox and shingles but also from virus shed from the respiratory tract. Virus inhaled by a susceptible contact replicates in the nasopharynx, spreading thereafter to cause the centripetal rash characteristic of chickenpox. Like other airborne virus infections, chickenpox is epidemic. Immunity is generally lifelong, with outbreaks mainly affecting susceptible birth cohorts. In temperate countries such as the United Kingdom and the United States, VZV is estimated to infect 60 to 90% of close and household contacts and, by age 10, more than 90% of the population are immune (1, 2). In contrast, VZV household infectivity in Guinea-Bissau, a tropical African country close to the equator, is closer to 16% (3). Unusually for a tropical climate, the mean age of chickenpox is similar to that of temperate countries, and this has been attributed to a higher population density which compensates for the reduced viral transmissibility (3). Possible explanations for the reduced infectivity of VZV in tropical countries include increased temperature, humidity, and UV light exposure, all of which have been shown *in vitro* to inactivate virus (4). However, prevalent viral genotypes circulating in Africa, India, and Sri Lanka differ from endogenous European genotypes, and this could also provide an explanation for different patterns of transmissibility (5). While more than 47 full-length VZV genomes have been sequenced to date (6–10), none are from viruses circulating in countries with low transmission rates.

Here, we have sequenced and assembled whole VZV genomes from 23 individuals over the course of a seasonal varicella outbreak in Guinea Bissau. These viruses were collected from a well-characterized population cohort in the Bandim peri-urban area of Bissau, the capital of Guinea-Bissau, which has been studied for over 30 years as part of the Bandim Health Project (Statens Serum Institut, www.bandim.org) (3, 11, 12). Epidemiological data were obtained for an outbreak of 1,419 cases occurring over a 7-month period during 2001, while samples of vesicle fluid were obtained from a subset of these (∼500). The data collected included house location, household structure (i.e., numbers of families and family members cohabiting), severity of disease, and the relationship of each infected person to the putative index patient who transmitted to them. While subgenomic regions (e.g., the origin of DNA replication [OriS]) undergo rapid changes during an epidemic (3), the extent to which new mutations accumulate across the whole genome during transmission is not known, and thus VZV transmission chains remain poorly characterized at the whole-genome level.

This study uses recently developed enrichment methods (13, 14) which enable deep sequencing of pathogens directly from clinical samples and is the first study of herpesviruses that investigates the origins and subsequent evolution of viral genotypes during an outbreak and provides unique insights into the biology of these large double-stranded DNA viruses. Finally, we address the long-standing question of whether varicella strains circulating in tropical countries have specific genetic adaptations that result in reduced transmission rates.

## MATERIALS AND METHODS

### Sample collection and ethics.

**FIG 1:**
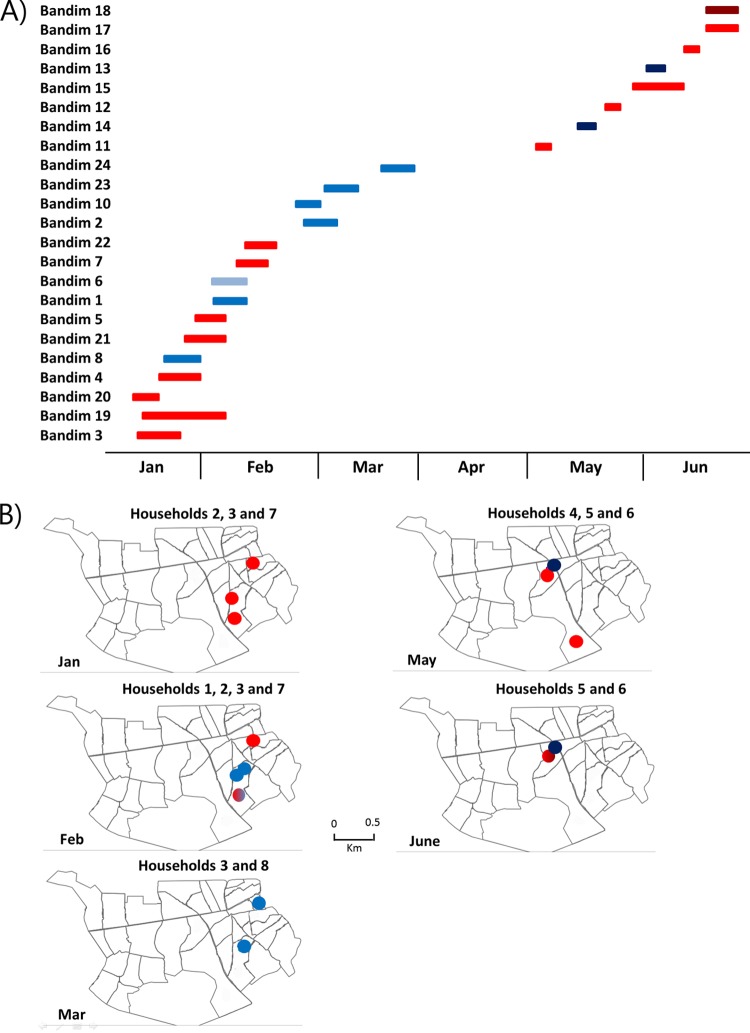
Geographic and temporal distribution of Bandim samples. Sampling of patients from eight households was performed within a 4-km^2^ area of the Bandim township during the start, middle, and end of a varicella outbreak. (A) Period of symptomatic infection for each patient. Data are colored according to the lineages described in Fig. 1 and 2. (B) Location of each sample household during the months in which sampling took place, again the data are colored according to lineage. Bisected circles indicated multiple lineages within a single household (e.g., household 1 during February).

Ethical clearance for the study was obtained from the Ministry of Public Health in Guinea-Bissau and the East London and City Health Authority. Participation was voluntary. We selected eight putative transmission chains comprising two to four members of the same household collected during the start, middle, or end of the outbreak in 2001 (Table 1 and Fig. 1). Putative transmissions were defined by the occurrence of a household member being diagnosed with varicella within 7 to 21 days of another member of the same household and where typing of the OriS region showed a similar number of TA and GA repeats (±1) (3). We also selected three samples (Bandim 6, 7, and 18) where the OriS repeat structure differed from other infected persons of the same household in the same time period. Vesicular virus was obtained from a total of 24 subjects with varicella during the outbreak, placed in viral transport medium and stored at −80°C.

**TABLE 1:** Overview of sample collection, household association, and putative transmission chains

Sample	Household	Age (yr)	Date of infection (day/mo)	Putative infection routes^*a*^			Accession no.
				Route A	Route B	Route C	
Bandim 1	1	7	7/2	Index			KM355696
Bandim 2	1	3	28/2	Recipient			KM355697
Bandim 3	2	9	15/1	Index			KM355698
Bandim 4	2	2	22/1	Recipient	Index		KM355699
Bandim 5	2	2	1/2	Recipient	Recipient		KM355700
Bandim 6	2	12	7/2		Recipient		KM355701
Bandim 7	3	2	12/2	Recipient		Index	KM355702
Bandim 8	3	6	24/1	Index	Index		KM355703
Bandim 10	3	NA^*b*^	1/3			Recipient	KM355704
Bandim 11	4	6	4/5	Index			KM355705
Bandim 12	4	2	21/5	Recipient			KM355706
Bandim 13	5	44	5/6	Recipient			KM355707
Bandim 14	5	1	14/5	Index			KM355708
Bandim 15	6	12	31/5	Index			KM355709
Bandim 16	6	4	11/6	Recipient	Index		KM355710
Bandim 17	6	10	18/6	Recipient	Recipient		KM355711
Bandim 18	6	4	18/6	Recipient	Recipient		KM355712
Bandim 19	7	8	17/1	Index			KM355713
Bandim 20	7	NA	17/1		Index		KM355714
Bandim 21	7	NA	30/1	Recipient	Recipient	Index	KM355715
Bandim 22	7	NA	15/2			Recipient	KM355716
Bandim 23	8	18	5/3	Index			KM355717
Bandim 24	8	1	21/3	Recipient			KM355718

a Up to three putative infection routes could be established per household given a transmission period of between 7 and 21 days after the onset of the first symptoms.

b NA, not available.

### DNA extraction, library construction, targeted enrichment, and sequencing.

Total DNA was extracted from each sample by using a QiaAMP DNA minikit (Qiagen) according to the manufacturer's instructions. DNA quantification was performed with a NanoDrop spectrophotometer, and samples with 260/280 ratios outside the range 1.7 to 2.1 and 260/230 ratios outside the range 1.8 to 2.2 were further purified using a Zymoclean Genomic DNA Clean & Concentrator (Zymo Research Corp.). Whole-genome amplification using GenomiPhi V2 (GE Healthcare) was performed using 10 ng of starting material. Libraries were constructed in accordance with the standard SureSelect XT v1.5 protocols (Agilent). Enrichment for VZV sequences was performed as described previously (13, 14). Sequence libraries were multiplexed (Bandim 1 to 12 and Bandim 13 to 24) and sequenced using 300-bp and 500-bp paired-end kits (respectively) on an Illumina MiSeq.

### Genome assembly and variant calling.

Sequence data set where demultiplexed using BaseSpace and individual data sets were subsequently parsed through QUASR (15) for duplicate removal and read-trimming (-q 30, -l 50) and subsequently aligned against the VZV reference strain Dumas (NC_001348) using BWA (16). Resulting alignments were processed using SAMTools (17) to generate pileup files for each sample. A consensus sequence for each data set was called with the QUASR module “pileupConsensus” and a 50% frequency threshold (i.e., no ambiguities were included). Variant profiling for each data set was performed using VarScan v2.2.11 (18) with the following parameters: basecall quality, ≥20; read depth, ≥50; and independent reads supporting minor alleles, ≥2 per strand. In addition, variant calls showing a directional strand bias of ≥0.85 were excluded from further analyses. Consensus sequences were generated for each rash sample, but iterative repeat regions R1, R2, R3, R4, and R5 (19, 20), as well as the terminal repeat region, were trimmed prior to tree-building analyses.

### Consensus sequence analyses.

DNA sequences were aligned by using the program Mafft, v6 (21), with alignments checked manually; no insertions or deletions were inferred from the alignment.

### Substitution rates.

Estimates of substitution rates were inferred using the program Beast v1.7.5 (22). The Beast analyses were performed under a HKY+I model of nucleotide substitution (selected by jModeltest 2.1 [23, 24]), strict and relaxed clock models (strict clock, relaxed lognormal, and relaxed exponential) and a variety or tree coalescent priors (constant, Bayesian skyline, and exponential). A Bayes factor analysis suggested that there was not sufficient evidence to reject a model of constant population size. The Monte Carlo Markov chain was run for 50,000,000 iterations, with a thinning of 50,000. We checked for convergence by ensuring that all parameters had an effective sample size (ESS) of at least 200. Finally, we assessed the molecular clock model by looking at the coefficient of variation histogram. The program Path-O-gen (http://tree.bio.ed.ac.uk/software/pathogen), which regresses the root-to-tip distance against the sampling date, was used to assess the “clock-likeness” (the extent to which the sampling date is correlated with the total branch length) of the Guinea-Bissau VZV sequence data using neighbor-joined trees inferred by Mega v5.2 (25). To assess the level of the temporal structure (which measures the effect that the background mutation rate is having on the inferred substitution rate) we adopted the approach of Duffy and Holmes (43) whereby rate analyses were repeated, but with the sampling dates randomly shuffled among the tips.

### Amplification of VZV reiteration regions.

The VZV reiteration regions R1, R2, R4, and R5 were amplified and sequenced from Guinea-Bissau sample DNA using primers designed against the Dumas reference genome (NC_001348) as a template. All PCRs were carried out using Herculase II fusion DNA polymerase (Agilent Technologies) according to the manufacturer's instructions. The cycling conditions were as follows: denaturation at 95°C for 2 min, followed by 35 cycles of amplification (denaturation at 94°C for 20 s, annealing at 55 to 65°C for 20 s, and extension at 72°C for 30 s), and then a final extension step at 72°C for 3 min. PCR products were purified using a DNA Clean & Concentrator-5 kit (Zymo Research) according to the manufacturer's instructions. Products were Sanger sequenced in the forward and reverse directions.

### GenBank accession numbers.

Consensus sequences for all samples sequenced in the present study are available in GenBank under the following accession numbers: KM355696 to KM355718.

## RESULTS

**FIG 2:**
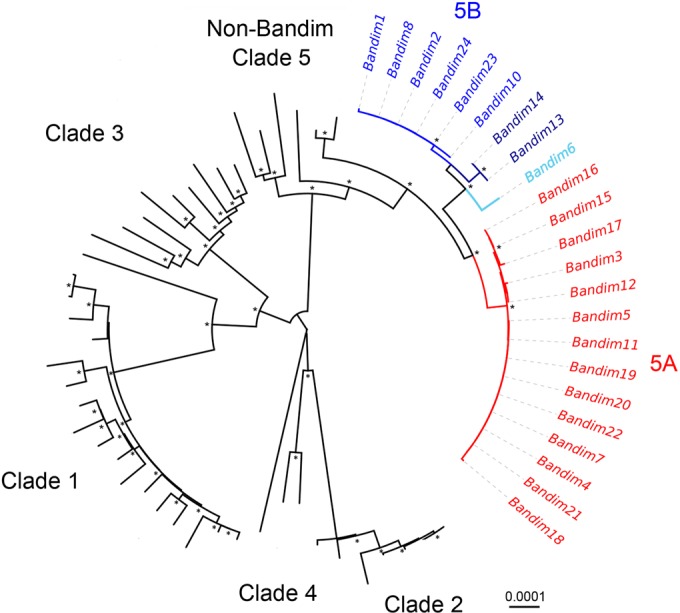
Neighbor-joined phylogeny identified two major clade 5 genotypes in Bandim samples. A neighbor-joined phylogeny (500 bootstraps) comprising all publicly available VZV genomes with the genomes presented here shows clear segregation of the major geographical clades, while the primary genogroups 5A and 5B segregate within clade 5. Bootstrap scores are not given where bootstrap values fall below 0.9. Well-supported nodes (>0.9) are indicated by an asterisk.

**FIG 3:**
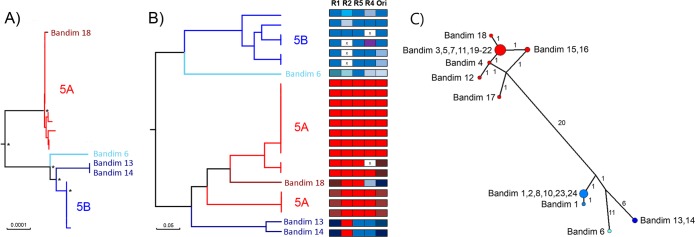
Phylogenies generated from full genome sequences and repeat region sequences only for Bandim sample collection. The neighbor-joined phylogeny comprising all Bandim samples (derived from Fig. 1) with asterisks denoting bootstrap support >0.9 (A) and a UPGMA (unweighted pair-group method with arithmetic averages) phylogeny (constructed from a distance matrix calculated from the repeat data where the variable is the number of repeat units) for the same sample collection using just the R1-R5 repeat region sequences (B) are shown. Repeat region patterns are identified by color and correspond to data shown in Table S1 in the supplemental material. (C) Phylogenetic network reveals multiple genotypes. Two primary genogroups are present in the Guinea-Bissau data set, while Bandim 6 and Bandim 13/14 are also considered different lineages. Nodes are colored according to the lineage (gray nodes are median joins), labeled according to the sample, and “sized” according to the number of identical genomes (not including variation in repeat regions). The numbers of SNP differences between consensus sequences are labeled along the branches (i.e., branch lengths are not scaled to number of changes).

**FIG 4:**
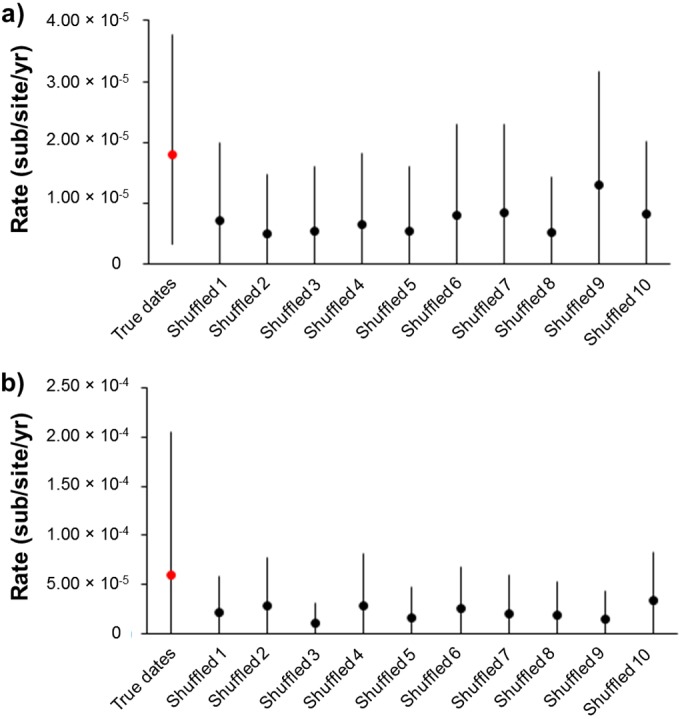
Estimates of VZV evolutionary rates. The evolutionary rate of VZV (mean and 95% highest posterior density [HPD]) is estimated from the true dates of sampling (highlighted in red) and from the shuffled dates of isolation (in black). The mean and 95% HPD interval is shown for each condition (the true and shuffled analyses). The results from either all 23 VZV whole viral genomes (a) or genogroup 5B alone (b) show that the estimated rate from both data sets overlaps with the 95% HPD intervals of the shuffled runs, indicating that there is little evidence of temporal structure.

VZV DNA was successfully enriched from 23 patient samples (Table 1), with over 99.9% genome coverage by Illumina MiSeq sequencing (Table 2). All 23 Guinea-Bissau viruses clustered within clade 5, and all Guinea-Bissau viruses were more closely related to each other than to other clade 5 viruses, although no single mutation differentiated them from other clade 5 viruses (Fig. 2). Virus sequences segregated into two main genogroups, 5A and 5B, and these cocirculated throughout the epidemic although no coinfected samples were found (Table 1 and Fig. 1 and 2). Most viruses within each genogroup were genetically nearly identical. However, three viruses in genogroup 5B (Bandim 6, 13, and 14) were more divergent than others compared to the consensus sequence for the genogroup. Bandim 6 differed from the consensus 5B sequence by 16 single-nucleotide polymorphisms (SNPs), and Bandim 13 and 14, although they were identical to each other, differed from the consensus 5B sequence by 11 SNPs (Fig. 3). The paired Bandim 13/14 viruses were recovered at the end of the outbreak and may therefore have evolved during epidemic spread. In contrast, Bandim 6 cocirculated with 5A and 5B viruses in the first month of the outbreak. Based on the timing of the second infections (within 7 to 21 days of the putative index case), we identified 15 potential household transmissions (Table 1 and Fig. 1). In nine of these, the SNP genotype of the transmitted virus sequence was identical to the index case, whereas in three they were identical apart from one (Bandim 1/2) or two SNP differences Bandim (11/12). However, for four putative household transmissions the viruses that appeared temporally linked were genetically distinct, indicating transmission from an external source (Bandim 4/6, 7/8, 16/17, and 16/18) (Fig. 4).

**TABLE 2:** Sequencing metrics for Bandim sample collection

Sample	No. of paired reads		%OTR^*a*^	MRD^*b*^	%COV^*c*^		OriS motif^*d*^	
	Total	Mapping			>1×	>100×	Sanger	NGS
Bandim 1	449,170	220,828	52.30	307	99.99	80.44	8-15	8-15
Bandim 2	479,705	425,669	91.96	583	99.99	99.21	8-15	8-15
Bandim 3	494,775	423,408	88.17	582	99.99	90.01	10-8	10-8
Bandim 4	477,905	431,040	92.53	589	99.99	96.83	10-8	10-8
Bandim 5	496,764	425,040	87.86	583	99.99	94.38	10-8	10-8
Bandim 6	605,683	424,736	73.13	577	99.99	93.99	9-12	9-12
Bandim 7	618,631	403,137	68.71	549	99.99	98.33	9-8	9-8
Bandim 8	622,270	480,279	79.65	659	99.99	94.12	**8-14**	**8-15**
Bandim 10	466,700	398,238	87.60	550	99.99	94.85	8-15	8-15
Bandim 11	508,867	453,839	91.97	623	99.99	99.38	**10-8**	**9-8**
Bandim 12	458,520	234,607	56.08	324	99.99	88.82	10-8	10-8
Bandim 13	1,198,584	946,813	80.84	1,955	99.99	97.47	9-15	9-15
Bandim 14	1,227,196	949,880	79.64	1,972	99.99	98.27	9-16	9-16
Bandim 15	1,410,853	1,209,428	87.69	2,471	99.99	97.02	9-9	9-9
Bandim 16	1,386,654	694,294	55.87	1,203	99.99	95.94	9-9	9-9
Bandim 17	891,210	619,377	74.58	848	99.99	95.07	9-9	9-9
Bandim 18	1,997,274	1,737,784	89.30	3,578	99.99	99.74	9-16	9-16
Bandim 19	1,714,100	1,479,316	88.37	3,074	99.99	98.74	10-8	10-8
Bandim 20	1,757,018	1,446,020	84.64	2,990	99.99	97.73	10-8	10-8
Bandim 21	845,990	745,560	89.61	1,543	99.99	97.89	10-8	10-8
Bandim 22	1,562,621	1,284,750	84.50	2,678	99.99	96.31	10-8	10-8
Bandim 23	1,428,708	1,213,904	86.75	2,444	99.99	99.69	8-14	8-14
Bandim 24	1,550,409	1,356,234	89.19	2,817	99.99	98.78	8-14	8-14

a %OTR, on-target read percentage (i.e., the percentage of VZV mapping reads).

b MRD, mean read depth per base.

c %COV, percentage of genome covered at defined read depth.

d The first number indicates the number of TA repeats, and the second number indicates the number of GA repeats. Boldfacing indicates where a discrepancy exists between Sanger and NGS data.

**FIG 5:**
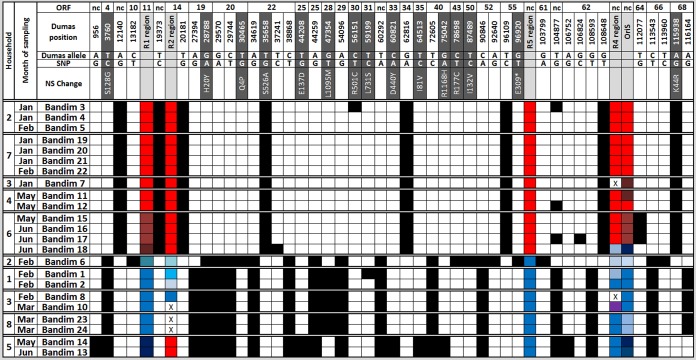
Forty-four SNPs segregate VZV genomes isolated during the 2001 outbreak. Black boxes indicate SNP differences from the Dumas reference genome (while empty boxes indicate agreement). The positions identified (nucleotide base and codon) are equivalent to Dumas (NC_001348). Nonsynonymous changes are highlighted in gray in the upper part of the table, while the repeat regions R1, R2, R4, R5, and the OriS are highlighted in purple. Red and blue shading of the repeat regions indicate the relative conservation of the repeat region sequences in each of the genogroups (e.g., R5 differs between genogroups but is perfectly conserved within each genogroup) and correlates with data shown in Fig. 3. Putative transmission chains are grouped (based on geographic information) in the first column with the month during which the sample was isolated shown in the second column. Repeat regions that could not be amplified are indicated by an “X” (note that R3 repeat regions are not included). nc, noncoding region; *, stop codon.

In total, 44 SNP positions were identified within our sample set, 15 of which are nonsynonymous mutations (Fig. 4). The majority of open reading frames (ORFs) contained either no SNP (*n* = 45) or a single SNP (*n* = 14). Four viruses—Bandim 6, 13, 14, and 17— had synonymous mutations at positions in ORF62. Three viruses—Bandim 15, 16, and 17—also shared a synonymous mutation in ORF64. Using the sampling dates for all 23 samples, we calculated the substitution rate (excluding the repeat regions) to be 1.82 × 10^−5^ substitutions per site per year (Tables 3 and 4). Estimates of the substitution rate were also obtained using strict clock models (where a single substitution rate applies to all parts of a phylogenetic tree) and relaxed clock models (where substitution rates are allowed to differ across the phylogenetic tree). Although these rates varied only slightly between strict and relaxed clock models, there was only weak evidence for temporal signal (a measure of the degree of clock-like [i.e., constant] evolution in the data) (Tables 3 and 4). This can be attributed to the cocirculation of multiple genotypes during the outbreak and thus we also calculated a substitution rate separately for genogroup 5B, for which there was better evidence for temporal signal (Tables 3 and 4). No substitution rate could be calculated for genogroup 5A since the genetic diversity between the samples was too low. Overall, these substitution rate calculations are higher than have previously been estimated for VZV (e.g., 3.8 × 10^−6^ substitutions per site per year [26]). To validate our estimate, the calculations were repeated after random shuffling of sampling dates between samples (27). Ideally, the mean evolutionary rate calculated from the true data should not coincide with the confidence intervals attached to the mean evolutionary rates calculated for any of the shuffled tips analyses (Fig. 4). However, some overlap was observed in our analyses, which suggests that the background mutation rate may be slightly inflating our estimate of the evolutionary rate. This is consistent with the short time scale over which the viruses in the present study were sampled and suggests that some of the variation observed between samples may be from deleterious mutations which are yet to reach fixation. To estimate the time of divergence of genogroups 5A and 5B, we used three different calculations of the substitution rate, the one derived here from all 23 samples (1.82 × 10^−5^ substitutions per site per year), one derived just from genogroup 5B samples (5.91 × 10^−5^ substitutions per site per year), and one obtained from a previous studies by Firth et al. (3.80 × 10^−6^ substitutions per site per year [26]). The dates of divergence, estimated from all three rates, for genogroups 5A and 5B, as well as the Bandim 6 and Bandim 13/14 lineages, are shown in Table 4 and Fig. 5.

**TABLE 3:** Substitution rates and estimates of time since the lineages diverged: clock model, mean evolutionary rate, and 95% highest probability density interval

Genogroup(s)	Clock model	Mean evolutionary rate (per site/yr)	95% HPD^*a*^ interval	
			Lower	Upper
5B and 5A	Strict	1.82 × 10^−5^	5.45 × 10^−7^	3.85 × 10^−5^
	Relaxed lognormal	2.19 × 10^−5^	1.66 × 10^−7^	5.08 × 10^−5^
	Relaxed exponential	2.65 × 10^−5^	1.05 × 10^−7^	5.81 × 10^−5^
5B only	Strict	5.91 × 10^−5^	3.86 × 10^−10^	2.05 × 10^−4^
	Relaxed lognormal	5.79 × 10^−5^	4.76 × 10^−9^	1.43 × 10^−4^
	Relaxed exponential	6.65 × 10^−5^	1.17 × 10^−7^	1.64 × 10^−4^

a HPD, highest probability density.

**TABLE 4:** Substitution rates and estimates of time since the lineages diverged: segregation, years since divergence, and 95% confidence interval

Genogroup rate used	Segregation	Time (yr) since divergence^*a*^	95% CI^*b*^	
			Lower	Upper
5B and 5A	5A and 5B	7.14	1.71	28.36
	5B and Bandim 6	3.47	0.86	13.70
	5B and Bandim 13/14	2.00	0.53	7.91
5B only	5A and 5B	2.29	1.68	2.95
	5B and Bandim 6	1.16	0.79	1.61
	5B and Bandim 13/14	0.81	0.56	1.12
Firth et al. (26)	5A and 5B	30.37	20.85	41.05
	5B and Bandim 6	14.21	8.26	21.24
	5B and Bandim 13/14	8.03	3.87	12.93

a That is, the time to the most contemporaneous tip (18 June 2001).

b CI, confidence interval.

In all but three cases, which differed by a single repeat unit (either TA or GA), the OriS sequences obtained by Illumina sequencing matched those previously obtained by PCR and Sanger sequencing (3) (see Table S1 in the supplemental material). The OriS and R4 repeat regions were present as two identical copies in all viruses (i.e., did not differ within a single sample but could still differ between samples). The OriS is the only region for which recombination could be postulated with a single genogroup 5A virus (Bandim 18) having a genogroup 5B-like OriS repeat structure. We were also able to amplify and sequence (by Sanger methodology) four of the five VZV tandem repeat regions (R1, R2, R4, and R5). Insufficient DNA remained to amplify the R3 repeat region. The repeat structures of the R2, R4, R5, and OriS sequences were similar to published sequences (see Table S2 in the supplemental material). However, the Guinea-Bissau viruses possess a hexamer repeat motif, termed ε, present at the 3′ end of the R1 repeat which has previously only been observed in four other clade 5 viruses (Table 5) and is currently considered to be unique to clade 5 viruses (9, 28). The hexamer repeat motif encodes a single aspartic and glutamic acid that serves to increase protein acidity at the C-terminal coiled/helical (end) region of the clade 5 R1 repeat.

**TABLE 5:** R1 repeat regions differ between clade 5 genomes and all other genomes

GenBank accession no./sequence identification	Clade	R1 motif^*a*^	Length (aa)
EU154348	1	[αββ][αββ][αββ][αβ][αβ][αβ]	81
JN704691	1	[αββ][αββ][αββ][αβ][αββ1][αβ]	86
AY548170	1	[αββ][αββ][αββ][αββ][αβ][αβ]	101
JN704693/NC001348	1	[αββ][αββ][αββ][αβ][βββ][αβδ][αβ]	86
DQ674250/AY548171/DQ479953	1	[αββ][αββ][αββ][αβδ][αβ][αβ]	97
DQ479962/DQ479963	1	[αββ][αββ][αββ][αβδ][αβ][αβ][αβ]	97
DQ479954	1	[αββ][αββ][αββ][αβδ][αββ][αββ][αβ]	107
JN704694	1	[αββ][αββ][αββ][βββ][αβδ][αβ]	90
DQ479961	1	[αββ][αββ][αβδ][αβ][αβ][αβ]	81
DQ479958/DQ479959	1	[αβ][βββ][αββ][αββ][αβ][α1βδ][αβ][αβ]	107
JF306641	2	[αββ][αββ][αββ][αββ][αββ][αβ]	91
AB097933	2	[αββ][αββ][αββ][δ][αβ][αβ]	75
AB097932/DQ008354/DQ008355	2	[αββ][αββ][αββ][δ][αβ][αβ][αβ]	86
JN704702	3	[αββ][αβ][ββ1β][αββ1][αβ]	69
JN704701	3	[αβδ3][αββ][αβ][αββ][αβδ3][αβ]	86
AJ871403/DQ479955/DQ479957	3	[αβδ3][αδ2β][αββ][αββ][αβ]	75
DQ479956	3	[αδ2β][αβ][αββ][αβ][αβ]	65
DQ479960	4	[αββ][αββ][αββ][δ][αβ][αβ][αβ]	86
DQ452050	4	[αββ][αβ][ββδ][αβ][ββδ][αβ][αβ][αβ]	101
Bandim 15/16/17	5	[αβδ1][αββ][αββ][αβ][εβ][εβ][εβ][εβ][εβ][εβ][εβ][εβ][εβ]	122
Bandim 18	5	[αβδ1][αδ2β][αββ][αβ][εβ][εβ][εβ]	80
Bandim 3/4/5/19/20/21/22/11/12/7	5	[αβδ1][αδ2β][αββ][αβ][εβ][εβ][εβ][εβ][εβ][εβ][εβ][εβ]	115
Bandim 8/10/1/2/23/24	5	[αβδ1][αδ2β][αββ][αββ][εβ][εβ]	78
Bandim 6	5	[αβδ1][αδ2β][αββ][αββ][εβ][εβ][εβ][εβ][εβ][εβ][εβ][εβ]	120
Bandim 13/14	5	[αβδ1][αδ2δ2β][αββ][αβ][εβ][εβ][εβ][εβ]	92
JN704705	5	[αβδ3][αββ][αββ][αβ][βββ][αβ][εβ][εβ]	99
DQ457052	5	[αβδ3][αδ2β][αββ][αββ][αββ][εβ]	87
JN704704	5	[αβδ3][αδ2β][αββ][αβ][βββ][αββ1][εβ]	97
JN704707	5	[αβδ3][αδ2β][αββ][αβ][βββ][αββ2][εβ]	97

a α, DAIDDE; β, GEAEE; δ, DAAEE; δ1, GETEE; δ2, GDAEE; ε, DE.

Two R5 alleles were observed that segregated completely by genotype and did not vary throughout the outbreak (Fig. 5). Although the other repeat regions appeared more variable, some evidence of conservation within each lineage was evident for R1, R4, and OriS, although less so for R2. (Fig. 3; see Table S2 in the supplemental material). OriS was the most variable, although two main alleles, segregating with 5A (8xTA/14xGA) and 5B (5xTA/9xGA), were evident. Of the 11 samples that differed from the consensus for their genogroup, five (Bandim 7, 11, 13, 23, and 24) differed by a single dinucleotide, of which four (Bandim 7, 11, 23, and 24) were otherwise identical. A single dinucleotide difference in OriS can occur even when resequencing the same sample and so may be considered an artifact in most cases. The six cases (Bandim 6 and Bandim 14 to 18) that differed by more than one dinucleotide repeat from the genogroup consensus coincided with six of the seven viruses that also had changes in R1 structure (Fig. 5). The remaining virus with changes in the R1 region had a single dinucleotide change in OriS (Bandim 13).

Together, these data are consistent with a pattern of lineage coevolution involving the R1 repeat region and the OriS (Fig. 5; see Table S1 in the supplemental material). In contrast, the R4 repeat region, which is noncoding and segregated according to lineage, does not vary or associate with the evolution of new lineages. Variation in the R2 repeat region located in ORF14, which codes for glycoprotein C, was lineage independent.

## DISCUSSION

We report here the first whole-genome sequencing of freely circulating uncultured VZV from an outbreak of chickenpox. The outbreak began in Bissau, the capital of Guinea-Bissau, at the beginning of January 2001 and ended 23 weeks later at the end of June 2001. All of the viruses sequenced belong to clade 5, which is thought to be endemic in Africa and parts of Southeast Asia. However, at least three distinct sublineages of 5A, 5B, and Bandim 6 were circulating during the first month of the outbreak, suggesting multiple viral origins. This finding corroborates a previous study that identified multiple clades among viruses cocirculating in time and geographical location during a chickenpox outbreak in the United Kingdom (29). We conclude that the most parsimonious explanation for this finding is that exposure to infectious virus from many sources is common and thus not a rate-limiting step to epidemic spread. Rather, as we and others have previously shown, it is probably the availability of sufficient susceptible hosts together with environmental conditions such as school gatherings (3). In the United Kingdom and United States, primary cases are typically school-aged children who become infected at school, and secondary infections are their cohabitants at home (1, 2). In Guinea-Bissau, the ages of primary and secondary cases do not differ, probably due to extensive mixing of preschool-aged children with older children both inside and outside the home (3). It was shown in the present study that the infectivity rate falls coincidentally with the school holidays and that mixing at school is an important facilitator of transmission. Primary infections caused by Bandim 6 and 17 are from patients of school age (12 and 10 years old, respectively), but those caused by Bandim 7 and 18 are not (representing 2- and 4-year-old patients, respectively). We therefore have insufficient data to conclusively state from our samples whether transmission facilitated by mixing at school is an important primary transmission route of infection into a household or not, and so the origin of the index viruses in this outbreak remains uncertain. The Bandim Health Project collects detailed epidemiological data about households, their inhabitants, their health, and recent travel. Questionnaires administered to households affected early in the outbreak failed to identify contact with herpes zoster or sporadic chickenpox, including cases imported from outside the area. Asymptomatic oral shedding of virus is well described (30–32) and, in the absence of evidence for contact with chickenpox or zoster, the possibility that orally shed live virus seeded this outbreak cannot be excluded.

**FIG 6:**
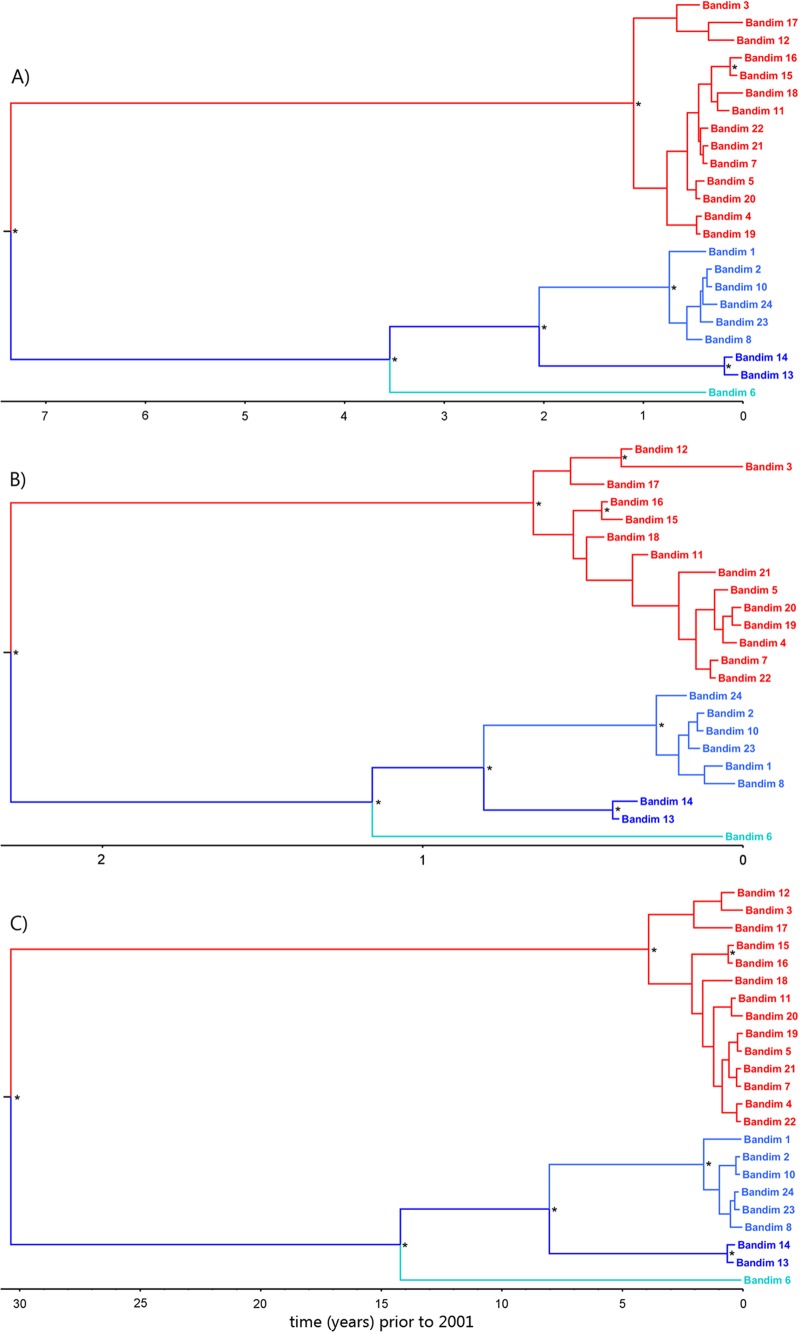
The major circulating genogroups and lineages diverged prior to the 2001 outbreak. Dated Bayesian phylogenetic trees showing the temporal relationships between VZVs sampled during the outbreak in Guinea-Bissau. Nodes with a posterior probability of >0.75 are indicated by asterisks, and the scale bar indicates time in years (i.e., since the date of the most contemporaneous sample (18 June 2001). Genogroup 5A is highlighted in red, and genogroup 5B is highlighted in blue. Dates were inferred with a strict clock (A), the rate fixed to that estimated from a strict clock analysis of just the genogroup 5B samples (B), and the rate fixed to that estimated from a previous study (26) (C).

Whole-genome sequencing confirmed VZV to be extremely stable during transmission (4, 8, 11); for example, 8 of the 14 genogroup 5A viruses (Bandim 4, 5, 7, 11, 19, 20, 21, and 22) were identical (barring one OriS dinucleotide repeat in Bandim 7 and 11) despite, in some cases, being recovered several months apart. A further three—Bandim 12, 15, and 16—differed by only a single SNP from the consensus sequence for their genogroup. There was more variation in the 5B genogroup, where at least two subsidiary divergent lineages appear to have arisen, at least one of which (Bandim 6) diverged prior to this outbreak. The substitution rates calculated from all of the sequences, excluding the repeat regions, and from just the 5B lineage, respectively, 1.82 × 10^−5^ and 5.91 × 10^−5^ substitutions per site per year, are significantly higher than previous estimates based on codivergence of the host and the virus (3.9 × 10^−9^ substitutions per site per year) (9) but only slightly higher than a previous estimate using heterochronous data (3.8 × 10^−6^ substitutions per site per year) (26). Previous analyses using time-stamped data in a range of other viruses have all inferred rates higher than that estimated by codivergence (33–36). Although the short sampling time may have inflated the estimates of substitution rates by including mutations that may be deleterious and become fixed over longer sampling times, the data are consistent with theories of VZV evolution that place the clade diversification of VZV some 20,000 to 50,000 years ago (37) rather than with the migrations out of Africa (26). Based on the substitution rates derived here and those derived by Firth et al. (26), we estimate that genogroups 5A and 5B diverged between 2 and 31 years prior to the outbreak, whereas the Bandim 6 lineage arose at least 1 and 14 years previously (Tables 3 and 4 and Fig. 6). We originally hypothesized that the Bandim 13/14 viruses, which were sampled toward the end of June, might have arisen by accumulation of mutations in genogroup 5B during the outbreak. However, even the fastest estimates of VZV mutation rate placed the date of divergence Bandim 13/14 from 5B as prior to the current outbreak (Tables 3 and 4 and Fig. 6). Sequencing of greater numbers of viruses from the outbreak is now required to corroborate (or refute) this finding. In addition, further sequencing should provide greater support for the mutation rate estimated here. The observation that length polymorphism in the R1, R4, and OriS is not random but rather lineage specific points to the possible coevolution of the R1 repeat region and the OriS (Fig. 6). However, no functional link between these two regions has been identified either in VZV or related herpesviruses. R1, which is located at the N terminus of the ORF11 protein (ORF11p), is upstream of a region of the ORF11p that has been predicted, *in silico*, to bind RNA (38–40). The R1 motif itself is predicted to form a hydrophobic alpha helix, which also typically binds to nucleic acid. It is unlikely that R1 directly binds to the OriS, since ORF11p is not known to be part of the DNA replication complex.

The structure and sequences of the Guinea-Bissau clade 5 repeat regions R2 to R5 and OriS overlap the repeat sequences found in other clades (see Table S2 in the supplemental material). The exception is the R1 tandem repeat, the C-terminal end of which contains densely repeating aspartic acid and glutamic acid residues, making it more acidic and hydrophobic than the R1 repeats found in clades 1 to 4 (see Table S2 in the supplemental material). ORF11 is expressed as an immediate-early protein in keratinocytes (18) and has been shown to be essential for replication in the SCID-Hu mouse skin xenograft model. As with its herpes simplex virus 1 homologue (UL47), the loss of ORF11p is associated with diminished expression of immediate-early proteins (38, 41, 42). Since clade 5 viruses are endemic to Africa and Southeast Asia (5), it is possible that the differences seen in clade 5 R1 represent adaptation to the host populations in these geographical regions. Further work is therefore needed to determine whether the R1 structure observed here is also found in clade 5 viruses from these regions and to determine whether this structure has an evolutionary or functional significance.

In summary, whole-genome sequencing of VZV has enabled us, for the first time, to study the dynamics of VZV transmission and evolution during a localized outbreak in Guinea-Bissau in 2001. These data have allowed us to accurately measure VZV short-term substitution rates and observe that all clade 5 viruses sequenced to date have a unique R1 repeat structure that codes for a more hydrophobic and acidic N terminus in the ORF11p. Guinea-Bissau clade 5 sequences cluster separately from clade 5 sequences obtained from European and U.S. subjects, suggesting a different evolutionary history, although sequencing of more African strains is needed to confirm this observation. Although most VZV remains highly conserved during epidemic transmission, our data imply that changes occurring in the R1 and OriS are associated with the evolution of new viral lineages.

## Supplementary Material

Supplemental material

## References

[1] Ross A Lenchner E Reitman G 1962 modification of chicken pox in family contacts by administration of gamma globulin. N. Engl. J. Med. 267:369–376. 10.1056/NEJM196208232670801. 14494142

[2] Hope-Simpson RE 1952 Infectiousness of communicable diseases in the household (measles, chickenpox, and mumps). Lancet ii:549–555. 10.1016/s0140-6736(52)91357-3 12981903

[3] Nichols RA Averbeck KT Poulsen AG al Bassam MM Cabral F Aaby P Breuer J 2011 Household size is critical to varicella-zoster virus transmission in the tropics despite lower viral infectivity. Epidemics 3:12–18. 10.1016/j.epidem.2010.11.003. 21420656 PMC3072572

[4] Rice PS 2011 Ultra-violet radiation is responsible for the differences in global epidemiology of chickenpox and the evolution of varicella-zoster virus as man migrated out of Africa. Virol. J. 8:189. 10.1186/1743-422X-8-189. 21513563 PMC3094303

[5] Breuer J Grose C Norberg P Tipples G Schmid DS 2010 A proposal for a common nomenclature for viral clades that form the species varicella-zoster virus: summary of VZV Nomenclature Meeting 2008, Barts and the London School of Medicine and Dentistry, 24 to 25 July 2008. J. Gen. Virol. 91:821–828. 10.1099/vir.0.017814-0. 20071486 PMC2888159

[6] Tyler SD Peters GA Grose C Severini A Gray MJ Upton C Tipples GA 2007 Genomic cartography of varicella-zoster virus: a complete genome-based analysis of strain variability with implications for attenuation and phenotypic differences. Virology 359:447–458. 10.1016/j.virol.2006.09.037. 17069870

[7] Peters GA Tyler SD Grose C Severini A Gray MJ Upton C Tipples GA 2006 A full-genome phylogenetic analysis of varicella-zoster virus reveals a novel origin of replication-based genotyping scheme and evidence of recombination between major circulating clades. J. Virol. 80:9850–9860. 10.1128/JVI.00715-06. 16973589 PMC1617253

[8] Grose C Tyler S Peters G Hiebert J Stephens GM Ruyechan WT Jackson W Storlie J Tipples GA 2004 Complete DNA sequence analyses of the first two varicella-zoster virus glycoprotein E (D150N) mutant viruses found in North America: evolution of genotypes with an accelerated cell spread phenotype. J. Virol. 78:6799–6807. 10.1128/JVI.78.13.6799-6807.2004. 15194755 PMC421634

[9] Zell R Taudien S Pfaff F Wutzler P Platzer M Sauerbrei A 2012 Sequencing of 21 varicella-zoster virus genomes reveals two novel genotypes and evidence of recombination. J. Virol. 86:1608–1622. 10.1128/JVI.06233-11. 22130537 PMC3264370

[10] Tipples GA Stephens GM Sherlock C Bowler M Hoy B Cook D Grose C 2002 New variant of varicella-zoster virus. Emerg. Infect. Dis. 8:1504–1505. 10.3201/eid0812.020118. 12498673 PMC2738511

[11] Poulsen A Cabral F Nielsen J Roth A Lisse IM Vestergaard BF Aaby P 2005 Varicella-zoster in Guinea-Bissau. Pediatr. Infect. Dis. J. 24:102–107. 10.1097/01.inf.0000151034.15747.4a. 15702036

[12] Poulsen A Qureshi K Lisse I Kofoed P-E Nielsen J Vestergaard BF Aaby P 2002 A household study of chickenpox in Guinea-Bissau: intensity of exposure is a determinant of severity. J. Infect. 45:237–242. 10.1053/jinf.2002.1049. 12423611

[13] Depledge DP Palser AL Watson SJ Lai IY-C Gray ER Grant P Kanda RK Leproust E Kellam P Breuer J 2011 Specific capture and whole-genome sequencing of viruses from clinical samples. PLoS One 6:e27805. 10.1371/journal.pone.0027805. 22125625 PMC3220689

[14] Depledge DP Kundu S Jensen NJ Gray ER Jones M Steinberg S Gershon A Kinchington PR Schmid DS Balloux F Nichols Ra Breuer J 2014 Deep sequencing of viral genomes provides insight into the evolution and pathogenesis of varicella-zoster virus and its vaccine in humans. Mol. Biol. Evol. 31:397–409. 10.1093/molbev/mst210. 24162921 PMC3907055

[15] Watson SJ Welkers MR Depledge DP Coulter E Breuer JM de Jong MD Kellam P 2013 Viral population analysis and minority-variant detection using short read next-generation sequencing. Philos. Trans. R. Soc. Lond. B Biol. Sci. 368:20120205. 10.1098/rstb.2012.0205. 23382427 PMC3678329

[16] Li H Durbin R 2009 Fast and accurate short read alignment with Burrows-Wheeler transform. Bioinformatics 25:1754–1760. 10.1093/bioinformatics/btp324. 19451168 PMC2705234

[17] Li H Handsaker B Wysoker A Fennell T Ruan J Homer N Marth G Abecasis G Durbin R 2009 The sequence alignment/map format and SAMtools. Bioinformatics 25:2078–2079. 10.1093/bioinformatics/btp352. 19505943 PMC2723002

[18] Koboldt DC Zhang Q Larson DE Shen D McLellan MD Lin L Miller CA Mardis ER Ding L Wilson RK 2012 VarScan 2: somatic mutation and copy number alteration discovery in cancer by exome sequencing. Genome Res. 22:568–576. 10.1101/gr.129684.111. 22300766 PMC3290792

[19] Davison AJ Scott JE 1986 The complete DNA sequence of varicella-zoster virus. J. Gen. Virol. 67:1759–1816. 10.1099/0022-1317-67-9-1759. 3018124

[20] Hondo R Yogo Y 1988 Strain variation of R5 direct repeats in the right-hand portion of the long unique segment of varicella-zoster virus DNA. J. Virol. 62:2916–2921. 2839710 10.1128/jvi.62.8.2916-2921.1988 PMC253729

[21] Katoh K 2002 MAFFT: a novel method for rapid multiple sequence alignment based on fast Fourier transform. Nucleic Acids Res. 30:3059–3066. 10.1093/nar/gkf436. 12136088 PMC135756

[22] Drummond A Suchard M Xie D Rambaut A 2012 Bayesian phylogenetics with BEAUti and the BEAST 1.7. Mol. Biol. Evol. 29:1969–1973. 10.1093/molbev/mss075. 22367748 PMC3408070

[23] Darriba D Taboada GL Doallo R Posada D 2012 jModelTest 2: more models, new heuristics and parallel computing. Nat. Methods 9:772. 10.1038/nmeth.2109. 22847109 PMC4594756

[24] Guindon S Gascuel O 2003 A simple, fast, and accurate algorithm to estimate large phylogenies by maximum likelihood. Syst. Biol. 52:696–704. 10.1080/10635150390235520. 14530136

[25] Tamura K Peterson D Peterson N Stecher G Nei M Kumar S 2011 MEGA5: molecular evolutionary genetics analysis using maximum likelihood, evolutionary distance, and maximum-parsimony methods. Mol. Biol. Evol. 28:2731–2739. 10.1093/molbev/msr121. 21546353 PMC3203626

[26] Firth C Kitchen A Shapiro B Suchard Ma Holmes EC Rambaut A 2010 Using time-structured data to estimate evolutionary rates of double-stranded DNA viruses. Mol. Biol. Evol. 27:2038–2051. 10.1093/molbev/msq088. 20363828 PMC3107591

[27] Duffy S Shackelton LA Holmes EC 2008 Rates of evolutionary change in viruses: patterns and determinants. Nat. Rev. Genet. 9:267–276. 10.1038/nrg2323. 18319742

[28] Norberg P Liljeqvist J-A Bergström T Sammons S Schmid DS Loparev VN 2006 Complete-genome phylogenetic approach to varicella-zoster virus evolution: genetic divergence and evidence for recombination. J. Virol. 80:9569–9576. 10.1128/JVI.00835-06. 16973560 PMC1617251

[29] Quinlivan M Sengupta N Papaevangelou V Sauerbrei A Grillner L Rousseva R Hague R Lutsar I Jogi P Leca A Grytchol R Alain S Breuer J 2013 Use of oral fluid to examine the molecular epidemiology of varicella-zoster virus in the United Kingdom and continental Europe. J. Infect. Dis. 207:588–593. 10.1093/infdis/jis649. 23087434 PMC3549596

[30] Nagel Ma Choe A Cohrs RJ Traktinskiy I Sorensen K Mehta SK Pierson DL Tyring SK Haitz K Digiorgio C Lapolla W Gilden D 2011 Persistence of varicella-zoster virus DNA in saliva after herpes zoster. J. Infect. Dis. 204:820–824. 10.1093/infdis/jir425. 21849278 PMC3156921

[31] Cohrs R Mehta S Schmid DS Gilden DH Pierson DL 2008 Asymptomatic reactivation and shed of infectious varicella-zoster virus in astronauts. J. Med. Virol. 80:1116–1122. 10.1002/jmv.21173. 18428120 PMC2938738

[32] Mehta SK Laudenslager ML Stowe RP Crucian BE Sams CF Pierson DL 2014 Multiple latent viruses reactivate in astronauts during space shuttle missions. Brain Behav. Immun. 41:210–217. 10.1016/j.bbi.2014.05.014. 24886968

[33] Shackelton LA Holmes EC 2006 Phylogenetic evidence for the rapid evolution of human B19 erythrovirus. J. Virol. 80:3666–3669. 10.1128/JVI.80.7.3666-3669.2006. 16537636 PMC1440363

[34] Lewis-Rogers N Bendall ML Crandall KA 2009 Phylogenetic relationships and molecular adaptation dynamics of human rhinoviruses. Mol. Biol. Evol. 26:969–981. 10.1093/molbev/msp009. 19182223

[35] Ramsden C Holmes EC Charleston MA 2009 Hantavirus evolution in relation to its rodent and insectivore hosts: no evidence for codivergence. Mol. Biol. Evol. 26:143–153. 10.1093/molbev/msn234. 18922760

[36] Harkins GW Martin DP Duffy S Monjane AL Shepherd DN Windram OP Owor BE Donaldson L van Antwerpen T Sayed RA Flett B Ramusi M Rybicki EP Peterschmitt M Varsani A 2009 Dating the origins of the maize-adapted strain of maize streak virus, MSV-A. J. Gen. Virol. 90:3066–3074. 10.1099/vir.0.015537-0. 19692547 PMC2885043

[37] Barrett-Muir W Scott FT Aaby P John J Matondo P Chaudhry QL Siqueira M Poulsen A Yaminishi K Breuer J 2003 Genetic variation of varicella-zoster virus: evidence for geographical separation of strains. J. Med. Virol. 70(Suppl 1):S42–S47. 10.1002/jmv.10319. 12627486

[38] Che X Oliver SL Sommer MH Rajamani J Reichelt M Arvin AM 2011 Identification and functional characterization of the varicella-zoster virus ORF11 gene product. Virology 412:156–166. 10.1016/j.virol.2010.12.055. 21276599 PMC3068617

[39] Che X Oliver SL Reichelt M Sommer MH Haas J Roviš TL Arvin AM 2013 ORF11 protein interacts with the ORF9 essential tegument protein in varicella-zoster virus infection. J. Virol. 87:5106–5117. 10.1128/JVI.00102-13. 23427162 PMC3624291

[40] Che X Reichelt M Sommer MH Rajamani J Zerboni L Arvin AM 2008 Functions of the ORF9-to-ORF12 gene cluster in varicella-zoster virus replication and in the pathogenesis of skin infection. J. Virol. 82:5825–5834. 10.1128/JVI.00303-08. 18400847 PMC2395146

[41] Zhang Y McKnight JL 1993 Herpes simplex virus type 1 UL46 and UL47 deletion mutants lack VP11 and VP12 or VP13 and VP14, respectively, and exhibit altered viral thymidine kinase expression. J. Virol. 67:1482–1492. 8382306 10.1128/jvi.67.3.1482-1492.1993 PMC237518

[42] Zhang Y Sirko DA McKnight JL 1991 Role of herpes simplex virus type 1 UL46 and UL47 in alpha TIF-mediated transcriptional induction: characterization of three viral deletion mutants. J. Virol. 65:829–841. 1846201 10.1128/jvi.65.2.829-841.1991 PMC239823

[43] Duffy S Holmes EC 2009 Validation of high rates of nucleotide substitution in geminiviruses: phylogenetic evidence from East African cassava mosaic viruses. J. Gen. Virol. 90:1539–1547. 10.1099/vir.0.009266-0. 19264617 PMC4091138

