# Supplementary material for: Evolution of Cocirculating Varicella-Zoster Virus Genotypes during a Chickenpox Outbreak in Guinea-Bissau
Source: J Virol. 2014 Dec;88(24):13936–46. doi: 10.1128/JVI.02337-14 (PMC4249134; doi:10.1128/JVI.02337-14)
Supplement: Supplemental material [file supp_88_24_13936__index.html]

Evolution of Cocirculating Varicella-Zoster Virus Genotypes during a Chickenpox Outbreak in Guinea-Bissau — Supplemental material 

# Evolution of Cocirculating Varicella-Zoster Virus Genotypes during a Chickenpox Outbreak in Guinea-Bissau

## Supplemental material

**Files in this Data Supplement:**

- Supplemental file 1 -

  Table S1 (R1, R2, R4, R5 and OriS repeat region motifs observed in the Bandim sample collection.)

  Table S2 (R1, R2, R4, R5 and OriS repeat region motifs observed in all Bandim samples and all samples derived from GenBank.)

  XLSX, 26K
